# Prediction of P53 Mutants (Multiple Sites) Transcriptional Activity Based on Structural (2D&3D) Properties

**DOI:** 10.1371/journal.pone.0055401

**Published:** 2013-02-13

**Authors:** R. Geetha Ramani, Shomona Gracia Jacob

**Affiliations:** 1 Department of Information Science and Technology, College of Engineering, Guindy, Anna University, Chennai, Tamilnadu, India; 2 Faculty of Information and Communication Engineering, Anna University, Chennai, Tamilnadu, India; Wake Forest University, United States of America

## Abstract

Prediction of secondary site mutations that reinstate mutated p53 to normalcy has been the focus of intense research in the recent past owing to the fact that p53 mutants have been implicated in more than half of all human cancers and restoration of p53 causes tumor regression. However laboratory investigations are more often laborious and resource intensive but computational techniques could well surmount these drawbacks. In view of this, we formulated a novel approach utilizing computational techniques to predict the transcriptional activity of multiple site (one-site to five-site) p53 mutants. The optimal MCC obtained by the proposed approach on prediction of one-site, two-site, three-site, four-site and five-site mutants were 0.775,0.341,0.784,0.916 and 0.655 respectively, the highest reported thus far in literature. We have also demonstrated that 2D and 3D features generate higher prediction accuracy of p53 activity and our findings revealed the optimal results for prediction of p53 status, reported till date. We believe detection of the secondary site mutations that suppress tumor growth may facilitate better understanding of the relationship between p53 structure and function and further knowledge on the molecular mechanisms and biological activity of p53, a targeted source for cancer therapy. We expect that our prediction methods and reported results may provide useful insights on p53 functional mechanisms and generate more avenues for utilizing computational techniques in biological data analysis.

## Introduction

Prediction of proteins, structures and methods to re-establish the normal state of activity in a biological structure is a significant task with profound social impact [1]–[2].Earlier studies on rescue mutants have detailed information reporting the results obtained using genetic strategies and p53 assays in the yeast and mammalian cells [1]. A number of human malignancies including lung, breast, head and neck, colorectal, pancreatic and gastric cancers confirmed the presence of high frequency of p53 mutations [1]–[6]. It was also reported that many malignancies detected at a young age could be successfully eradicated even in highly advanced stages [1] [6]–[7]. Moreover re-establishing wild type p53 function would benefit a large sector of cancer victims by providing ample scope for therapy [7]–[8]. In-vitro experimentation of each mutation site and patient record is a labour- and resource –intensive task consuming voluminous quantity of time, expertise and capital [1] [7] [9]–[10]. In view of this, we believed there was adequate justification to carry out a detailed exploration on the use of computational techniques to investigate the occurrence and activity of p53 mutants that could further lead to novel measures of developing therapeutic remedies from the structure and functional mechanism of cancer rescue mutations.

P53, also known as TP53 or tumor protein or tumor suppressor p53 is a tetramer multi domain transcription factor that has an essential role in maintaining the genomic integrity of the cell by controlling the cell cycle and inhibiting the formation of tumours [1]–[2] [11]–[13]. Wild-type p53 negatively regulates cell growth and division, whereas p53 mutants do not suppress cell growth and in some cases promote the growth of tumour cells [14]–[16]. In nearly half of all human cancers, this inactivation was an obvious consequence of mutations in the p53 gene [16]–[18]. However previous research and reports have affirmed the fact that loss of p53 activity due to missense mutations at the core DNA Binding Domain (DBD) could be restored by second site suppressor mutations [1] [12] [17]. Considering the cost of labour and resources involved in in-vitro experimentation of p53 mutations, it was highly essential and imperative to formulate computational strategies and techniques to analyze the consequences of diverse mutations and detect pertinent features that reinstated inactive (non-functional) mutations to active (functional) state.

Previous work on p53 mutant transcriptional activity prediction is attributed to Mathe et al. [19]who reported a Residual Score Profile (RSP) predicted transactivation accuracy varying from 64.2% to 78.5% using decision –tree models on missense mutants obtained from the Protein Data Bank. Recent work on multiple-site p53 transcriptional activity was carried out by Huang et al., [20] in which the authors used eight types of features to represent the mutants and then selected the optimal prediction features based on the maximum relevance, minimum redundancy (mRMR) approach [21], and Incremental Feature Selection (IFS) method. The Mathew’s Correlation Coefficient (MCC) [22] obtained by using Nearest Neighbour (NN) algorithm [23]–[24] and jack-knife cross validation [22]for one-, two-, three- and four-site p53 mutants were 0.678, 0.314, 0.705, and 0.907, respectively. Their investigation however did not include five-site and six-site p53 mutants and the authors have not reported on the performance of other standard feature selection or classification algorithms.

In order to portray the impact of applying computational techniques in predicting clinical outcomes, the current investigation focussed on the recent article by Huang et al. [20] published in this journal that reported the MCC of Nearest Neighbor algorithm on predicting p53 mutant transcriptional activity by means of Incremental Feature Selection with the mRMR method. We chose this paper for three main reasons. First, their work is the most recent and the data is publicly available to replicate the work. Second, p53 mutants are a great challenge to both biological and computer science researchers because of their imbalanced class distributions and voluminous records. Third, their work presented both biological and computational advancement that led researchers to focus on specific regions in the p53 core domain that significantly influenced p53 activity. However their results did not support a comparative study of classifier performance and focussed only on the predictive power of the NN algorithm. Moreover they had introduced a novel predictor approach to predict all types of mutation records irrespective of the nature of records (class distribution), number of instances and type of mutation (independent/co-occurring). We believed it was quite unlikely that a single predictive technique be able to classify well such diverse nature of data.

This research was dedicated to formulating novel computational approaches to predict and classify the transcriptional activity of multiple site (one-site, two-site, three-site, four-site, and five-site) p53 mutants using optimal set of predictive features that generated higher MCC and accuracy in prediction compared to previous work. Our method placed emphasis on the 2D structure surface of the p53 mutants and the 3D structural changes of the tumor protein, that have been reported to be highly essential in deciding the p53 activity [20]–[21] [25]. In this work we introduced three novel predictor methods. The first method targeted the detection of single independent p53 mutation activity while the second and third approaches were found suitable to predict the activity of co-occurring mutations that combined with the one-site p53 mutants. The second approach generated higher MCC in prediction with both a very large/small number of instances and imbalanced class distribution of records while the third approach served well with fewer instances and balanced records. To maintain brevity, we will call the first, second and third approaches as Independent Predictor (IP), Imbalanced Mutation Predictor (IMP) and Balanced Predictor (BP) methods respectively. We utilized the feature sets obtained by the CFS Subset Evaluator commonly for all the approaches. The features extracted by this technique were passed in an incremental manner to the classifiers to determine the prediction accuracy. Three benchmark classification algorithms viz, Bayesian Network Learning algorithm and Ensemble classifiers viz, AdaBoost Learning using Decision Stump (ABDS) algorithm and Random Committee using Random Tree (RCRT) algorithm showed improved results in prediction. The performance of the classifiers was evaluated using Jack-knife cross-validation technique based on the following scores: Mathews Correlation Coefficient 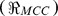, Accuracy 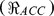, Sensitivity 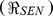 and Specificity 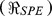. We also establish the fact that the utilization of 2D and 3D structural details of the p53 mutants showed higher prediction accuracy in detecting the p53 mutant transcriptional activity. It has also been validated by analysis of the feature sets that 2D structure features constituted a substantial portion of the optimal feature sets and played a pivotal role in transcriptional activity prediction of p53 site-specific mutations.

Previous, recent and related research on p53 mutants, Cancer and computational approaches have reported that the following requirements [25]–[26] be met for a successful predictor for biological data. They are stated to be the need for an authenticate, standard dataset to train and test the predictor, formulation of suitable statistical/scientific expressions that rightly signalled the inherent association of the predictor features with the target attribute, the existence of an algorithm or system that performed the prediction followed by the statement of evaluation measures to rank the estimated accuracy of the predictor [27]–[28]. We deal with the aforementioned methodology in the following sections.

## Materials and Methods

### Dataset

The P53 Mutant dataset available at the University of California, Irvine (UCI) Machine Learning (ML) Repository that can be accessed at http://archive.ics.uci.edu/ml/p53Mutants [29]–[32] was utilized as the benchmark dataset to train and test the proposed predictor system. Biophysical models of mutant p53 proteins yielded the features to predict the transcriptional activity. All class labels were determined via in vivo assays [31]. There were a total of 5409 attributes per instance. The attribute description is provided as Table S1. Attributes named V1–V4826 represented 2D electrostatic and surface based features. Attributes V4827–V5408 represented 3D distance based features. The target attribute was denoted by V5409 that carried two possible values to represent p53 transcriptional activity (Active/Inactive). The dataset initially comprised of 16772 p53 mutant records. This was primarily analyzed to filter the records that could not be encoded (records held missing values specific to 2D/3D structural properties). This resulted in the removal of 180 instances thus reducing the total size of the data to 16592 records. The data was further partitioned to identify the structural features pertaining to specific secondary-site mutations resulting in 5 subsets as depicted in Table 1.

**Table 1:** Site-Specific P53 Mutant Records.

*S.No*	*Site*	*Active Records*	*Inactive Records*	*Total No.of Records*
1.	1	8	54	62
2.	2	57	16319	16376
3.	3	63	49	112
4.	4	7	24	31
5.	5	6	2	8
Training records				16589

Once the data was pre-processed to suit the software specifications, the computational techniques were explored to generate the prediction of the p53 transcriptional activity. The preliminary requirement as mentioned by Huang et al. [20] was said to be the formulation of peptide samples with a potential mathematical relation to design an effective predictor system. The expression needed to significantly portray the intrinsic correlation of the predictor with the target to be predicted. The set of predictors was given by the following relation
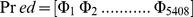
(1)where the subscript reflected the dimension of the vector and its value, while the components 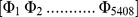 were defined by a series of features as elaborated below.

### 2D Structure Features

The 2D features were also known as the Surface Property Maps. The structure features for each mutant were obtained using the homology models described in [31]–[32]. The structures of mutant proteins were simulated centred on the configuration of wild type p53 by substitution with mutant amino acids following which the structure features were extracted from the energy minimized mutant model [30]. The 2D surface property maps were annotated with surface properties, such as electrostatics or h-bond donor/acceptor status provided by the electrostatic add-ons to AMBER 6 by Luo et al. [33].The molecular surface was mapped to a sphere, following which steric and depth information was recorded, and the sphere was mapped to a plane. The resulting surface map was subtracted from the wild-type map to obtain the resulting 2D features. The attributes 1–4826 of structure features (V1–V4826) were calculated based on the 2D surface map of the mutant protein [30]–[32].

### 3D Structure Features

3D features were also termed the Structure Distance Maps. Attributes 4827–5408 (V4827–V5408) of structure features were calculated based on the 3D distance difference map between mutant and wild-type p53 [30]–[32]. Mutation of amino acid in p53 could be responsible for alteration in the protein 3D structure. The 3D distance map was an N×N matrix giving the Cartesian distance between N residue alpha carbons. It reflected structural shifts induced by the mutation [32]. The wild-type distance map was subtracted, leaving a difference map. The p53 core domain had 197 residues, hence resulted in a 197×197 matrix that was collapsed to a distance vector that gave the magnitudes of the distance changes [32]. This resulted in a 197 length vector map portraying three features for each residue, the directional i, j, and k vectors. This summed up to 591 features per mutant of which 582 features alone were retained as significant attributes [31]–[32]. The 3D distance difference map features symbolized the magnitudes of the distance changes in the 3D structure [30]–[32].

Both the 2D structure features and 3D structure features were downloaded from the UCI Machine Learning Repository [29] and their annotations are supplied as Table S1.Thus a total of 5408 features constituted the attribute (feature) vector for each record in the p53 mutant dataset while attribute 5409 indicated the target category.

### Record Space of Site-specific Mutants

Table 1 depicts the breakdown of the number of records reflecting the total number of active and inactive records in each site-specific subset. There were a total of 5 subsets, partitioned based on the primary and co-occurring mutations sites.

### General Computational Approach

**Figure 1:**
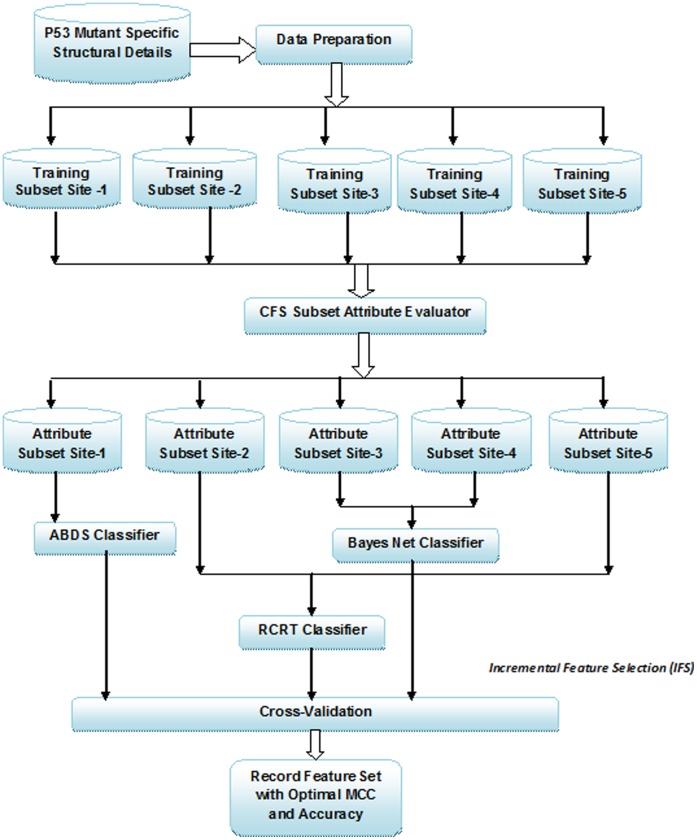
Novel Computational Approach to Predict Site-Specific P53 Mutant Transcriptional Activity.

The proposed approach for p53 mutant transcriptional activity prediction through computational approaches is portrayed in Figure 1. The approach comprised of the Training phase followed by the Prediction Phase. The former involved preparing the mutation data for process on software tools, data partitioning, and relevance detection of each attribute in the partitioned subset, construction of the prediction models and generation of prediction rules pertaining to each site. The SPSS software [34] was used to visualize and pre-process the mutant structural details according to the mutation sites. Data are available at www.shomonagjacob-research.com containing structural properties of site-specific mutations. The computational analysis of the data was done with data recorded on Microsoft Excel’s Comma Separated Version files. The Machine Learning Tool WEKA [35] was employed to perform attribute subset evaluation of the attributes using the CFS subset attribute evaluator algorithm [35]. The classification techniques utilized to build the predictor models for the mutation sites with the generated feature sets were also implemented in WEKA. The Prediction phase involved validation of the prediction accuracy and performance evaluation of the classifier. Jack-knife cross-validation [36] was employed wherein each of the statistical samples was taken to be the test case with the remaining samples considered the training set. The average MCC, accuracy, sensitivity and specificity was recorded to rate the performance in prediction of p53 mutant transcriptional activity and identify the classification algorithm that generated the highest MCC. The description of the attribute evaluator (feature ranking) and classification techniques in the proposed predictor methods are detailed below.

### Correlation Feature Selection (CFS) Subset Attribute Evaluator Method

Feature Selection [21] [23]–[24] played a crucial role in classifier design as several reports [20]–[26] have previously affirmed with acceptable justification. The most important phase in construction of classifiers was to identify the most representative set of predictor attributes [37]–[38]. The CFS hypothesis [35] [39] suggested that the most predictive features needed to be highly correlated to the target class and least relevant to other predictor attributes.

The following equation dictated the merit of a feature subset S that consisted of ‘k’ features [40]–[41]:
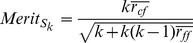
(2)where, 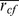 was the average value of all feature-classification correlations, and 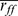 was the average value of all feature-feature correlations. The CFS criterion [39]–[41] was defined as follows:
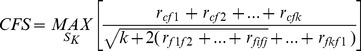
(3)where 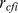
and 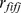
variables are referred to as correlations. The attributes that portrayed a high correlation to the target class and least relevance to each other were chosen as the best subset of attributes [42].

The attributes filtered by the CFS Subset Evaluator method were added in an incremental manner to identify the optimal set of features that contributed to prediction of p53 activity. This methodology is reported below.

### Incremental Feature Selection (IFS) Method

Utilizing the predictor attributes reported by the CFS Subset Attribute Evaluator method, Incremental Feature Selection (IFS) [43]–[51] was applied to determine the minimal and optimal set of features. The predictors generated by the CFS Subset evaluator were the feature set under consideration for Incremental Feature Selection. On adding each feature, a new feature set was obtained and the n^th^ feature set could be stated as
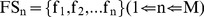
(4)Where M denoted the total number of predictor subsets. On constructing each feature set, the predictor model was constructed and tested through Jack-knife cross-validation method. The MCC/Accuracy of cross-validation was measured, leading to the formation of the IFS table with the number of features and their performance. FS_mo_ was the minimal and optimal feature set that achieved the highest MCC for each site-specific mutation.

The novel predictor methods proposed for the site-wise mutation activity prediction are detailed below.

### Independent Predictor Method

The independent predictor method applied to detecting the transcriptional activity of one-site mutations based on their 2D and 3D structural features. The method involved attribute evaluation by CFS Subset evaluator method followed by Incremental Feature Selection to determine the predictive accuracy of the classifier. The Adaboost algorithm using the Decision Stump (ABDS) prediction technique was utilized to categorize the functional activity of p53 one-site mutations. The algorithm execution used 100 iterations to obtain the most reliable results. The algorithms are briefed about in the following sections.

### Decision Stump Algorithm

The Decision Stump algorithm was introduced by Wayne Iba and Pat Langley in 1992 [52]. It was a machine learning model that generated a single level decision tree that comprised of a single node connected to the leaf nodes [52]–[53]. The decision stump made a prediction based on the value of just a single input feature. In the case of data that contained continuous values, a threshold feature value was selected, and the stump contained two leaves, one for values below and the other for values above the threshold. Multiple thresholds when chosen lead to generation of more leaf nodes. Decision stumps have been widely used as components (weak learners/base learners) in machine learning ensemble techniques like boosting [54]–[55].

### Adaboost Ensemble Learning with Decision Stump Method (ABDS)

Adaptive Boosting (AdaBoost), a machine learning algorithm, was formulated by Yoav Freund and Robert Schapire [56]–[57]. AdaBoost, a meta-algorithm, was used in conjunction with many other learning algorithms to improve their performance. AdaBoost was adaptive such that subsequent classifiers built were modified in favour of those instances misclassified by previous classifiers. Adaboost, an ensemble method of prediction used a combination of models [58]. Each combined a series of ‘k’ learned models with the aim of creating a composite model. Initially, Adaboost assigned each training instance an equal weight that equalled 1/number of training instances [56]. Later ‘k’ classifiers were generated that required ‘k’ rounds. In each round, instances from the dataset were sampled by weight to form the training set [55]–[56]. A classifier model was derived and its error rate was computed with the training set that later served as the test set. The instance weights were adjusted according to the error-rate. Records correctly classified were weighed less while misclassified records were made to weigh more [58]. Those weights were considered to generate the training tuples for the subsequent round. The error-rate of a generated model M_k_, was computed as follows [56]–[58]:
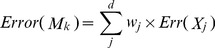
(5)Where Err(X_j_) was the misclassification rate of instance X_j_. If it was a misclassified instance the value was 1 else it was 0. If the performance of the classifier M_k_ was very poor (>0.5), it was abandoned and a new training set was generated to construct a new classifier model. If an instance was correctly classified in round ‘k’, its weight was updated by



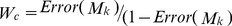
(6)The weights of all the correctly classified instances were updated likewise while the weights of the unclassified tuples were normalized to restore their sums to the initial value. Normalization was done by multiplying it by the sum of the old weights divided by the sum of the new weights.

After generation of the classifier ensemble, boosting assigned a weight to each classifier’s vote based on its performance. The weight of a classifier’s (M_k_) vote [56] [58]was given by
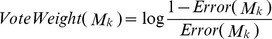
(7)


For each class, the sum of the weights of each classifier that assigned class c to an instance ‘X’ was determined. The class with the highest sum was considered as the category of the instance X.

The predictor method for imbalanced set of mutant data is discussed in the ensuing section.

### Imbalanced Predictor Method

The imbalanced predictor method was applied to mutation data that contained either too few or very large number of instances. This prediction technique comprised of attribute evaluation via CFS Subset evaluator followed by Incremental Feature Selection with the Random Committee Ensemble classifier with Random Tree (RCRT) algorithm. The algorithms are discussed below.

### Random Tree Classifier

Random trees were first introduced by Leo Breiman and Adele Cutler [59]. Random trees referred to a collection (ensemble) of tree predictors [60].The input feature vector was given to the classifier that classified it with every tree in the forest, and output the class label that received the majority of votes (weights) [61]. All the trees were trained with the same parameters, but on different training sets, that were generated from the original training set using the bootstrap procedure, i.e., for each training set vectors were selected randomly that equalled the number in the original set [62]. The vectors were chosen with replacement, i.e., some vectors occurred more than once and some did not occur at all. At each node of each tree trained, only a random subset of the nodes was used to identify the best split [63]–[64]. With each node a new subset was generated, whose size was fixed for all the nodes and all the trees. This referred to the training parameter denoted by √number of variables. In random trees the error was estimated internally during the training phase [63].

### Random Committee with Random Tree Classifier (RCRT)

The Random Committee generated an ensemble of classifiers for any base classifier that executed the Randomnizable Interface [35] [37]. We utilized the RCRT approach that constructed an ensemble of classifiers with Random Tree as the base classifier [64]. The random committee algorithm raised a diverse ensemble of random tree classifiers [65]. The random committee algorithm generated predictions by averaging probability estimates over the generated classification trees.The final prediction was a straight average of the predictions generated by the individual base classifiers [64]–[66].The algorithm was implemented in WEKA [35] with default parameters.

The prediction techniques that generated higher MCC for prediction of balanced and acceptable number of mutant records are given below.

### Balanced Predictor Method

Our investigations revealed that the number of mutation records and the class balance did play a pivotal role in deciding classifier results. Hence we attempted to compare three benchmark classification techniques to identify the algorithms that generated higher MCC and accuracy in prediction with the CFS Subset Evaluator attributes on data that contained balanced records. Our comparisons revealed that the Bayesian Network algorithm generated a higher MCC and accuracy than previously reported results on classification of site-3 and site-4 mutation data with 112 and 31 records respectively, much higher that the site-5 data subset and much smaller than the site-2 data subset. This predictor method employed the features returned by the CFS Subset evaluator method with the Bayesian Network Learning Algorithm.

### Bayesian Belief Network Learning Algorithm

A Bayesian network was a probabilistic graphical model/statistical model that represented a set of random variables and their conditional dependencies via a directed acyclic graph (DAG) whose nodes represented random variables [67]–[68]. The edges represented conditional dependencies while unconnected nodes represented variables that were conditionally independent of each other. Each node was associated with a probability function that took in as input a particular set of values for the node's parent variables and gave the probability of the variable represented by the node [69]–[70]. In this research, we utilized the Bayesian network to model the relationship between structural properties of mutants and their functional activity. Given the structural details, the network was used to compute the probabilities of the possible functional activity (active/inactive).

The learning task consisted of finding an appropriate Bayesian network given a data set D over U where U = {u_1_, u_n_}, n ≥1 was the set of input variables [67] [69]. The classification task consisted of classifying a variable y = x_0_ called the class variable (active/inactive) given a set of variables U = u_1_... u_n_. A classifier C: u → y was a function that mapped an instance of u to a value of y. The classifier was learned from a dataset D that consisted of samples over (u, y) [68]. A Bayesian network over a set of variables U was a network structure B_s_, a directed acyclic graph (DAG) over the set of variables U and a set of probability tables given by
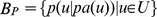
(8)


Where pa(u) was the set of parents of u in B_S_ and the network represented a probability distribution given by
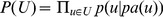
(9)


The inference made from the Bayesian Network was to allocate the category with the maximum probability [70]–[71]. The Simple Estimator with the K2 local search method using Bayes Score were utilized (default parameters) for the execution of the algorithm in WEKA [35].

The performance evaluation methods and parameters are briefed about in the subsequent section.

### Jack-knife Cross-Validation Method

Statistical prediction methods generally involved verification of the predictor performance to estimate their effectiveness in practical applications [72]–[73]. Cross-validation (rotation estimation), was a technique that assessed how the results of a statistical analysis could generalize to an independent data set. It was a way to predict the fit of a model to a hypothetical validation set when an explicit validation set was not available [72]–[73]. In *k*-fold cross-validation, the original sample was randomly partitioned into *k* equal size subsamples. Of the *k* subsamples, a single subsample was retained as the validation data for testing the model, and the remaining (*k* –1) subsamples were used as training data [73]. The cross-validation process was then repeated *k* times with each of the *k* subsamples used exactly once as the validation data. The *k* results from the folds were later averaged to produce a single estimation. In this study, the jack-knife cross validation method was used for validation since previous reports have stated it to be least arbitrary in nature and widely recognized by researchers to assess the performance of predictors [20] [72]–[73]. In jack-knife cross-validation, each one of the statistical samples in the training dataset was in turn singled out as a tested sample and the predictor was trained by the remaining samples. During the jack-knifing process, both the training dataset and testing dataset were actually open, and a statistical sample moved from one set to the other [20]. However since the second site mutations held voluminous records, in order to reduce the memory effects and computational complexity we used the three-fold cross-validation technique to rate and compare the performance of the prediction techniques. Moreover the analysis of the second site p53 mutation dataset exposed heavy imbalance of the active and inactive records. In view of this, the following indexes were adopted to test our proposed predictors.
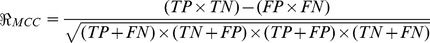
(10)

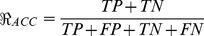
(11)

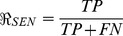
(12)

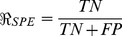
(13)where 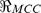
reflected the Mathews Correlation Coefficient; 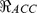
reflected the accuracy, i.e., the rate of correctly predicted mutation activity; 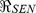 reflected the sensitivity, i.e., the rate of inactive records correctly predicted; 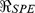 reflected the specificity, i.e., the rate of active records that were correctly predicted.

TP, TN, FP and FN denoted the number of true positives, true negatives, false positives and false negatives, respectively [20] [22]. However the MCC parameter was believed to estimate more precisely the performance of a predictor model on heavily imbalanced data and hence was given precedence.

## Results

The results of the proposed predictor models are discussed in three sections. The first section presents the performance of the attribute evaluators. The second section portrays the optimal performance of the three proposed predictor models. The third section depicts the comparative performance of the attribute evaluator and classification techniques analysed in this study.

### CFS Subset Attribute Evaluation Results

The feature set size filtered by the attribute evaluator techniques are tabulated in Table 2. The CFS Subset Evaluator, Information Gain, Gain Ratio and Symmetric Uncertainty Attribute Evaluators were compared in this work. It is evident from the results that the minimal feature set was generated by the CFS Subset Evaluator. Hence focus was placed on exploiting this technique to build predictor models with the minimal set of predictive features. Moreover the rank and score of the predictors generated by the other predictor models were more often negligible and hence their contribution to the prediction was questionable. Combining the CFS Subset Evaluator with the feature ranking methods was found to be very time-consuming and computationally expensive since the data spanned large number of attributes. On smaller datasets, the results showed only marginal variation. In the case of the site –two mutation data, the CFS subset Evaluator was applied on subsets of the mutation records with the 2D and 3D features being considered separately for analysis in order to speed up the execution process. Since the evaluator method filtered attributes with respect to its contribution to the target class and relevance to the other attributes, the cumulative results of the subset data were taken as the minimal feature set for the site-two predictor model. The CFS Subset Attribute Evaluator results for site-specific mutant data are provided as Table S2.

**Table 2:** Performance of Attribute Evaluator Algorithms on Site-Wise P53 Mutants Transcriptional Activity.

*S.No*	*Site*	*Number of Selected Features*			
		*CFS Subset*	*Information Gain*	*Gain Ratio*	*Symmetric Uncertainty Evaluator*
1	One	11	19	19	19
2	Two	52	50	40	40
3	Three	35	417	417	417
4	Four	16	73	73	73
5	Five	154	154	154	154

### Incremental Feature Selection Results

**Figure 2:**
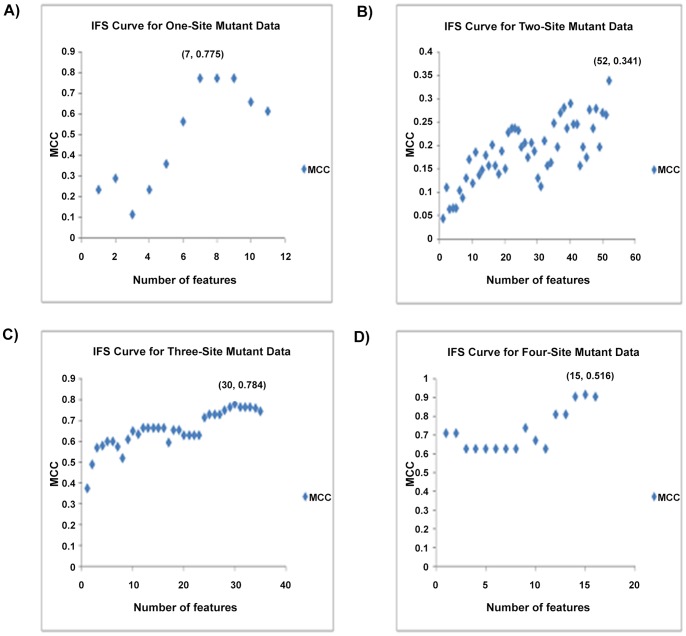
The IFS Curves for one-site, two-site, three-site, and four-site p53 mutants. In the IFS curve, the x-axis is the number of features used for classification, and the y-axis is the Mathew’s correlation coefficients (MCC). (A) The IFS curve for one-site p53 mutants. The peak of MCC is 0.775 with 7 features. The top 7 features derived by the CFS Subset Evaluator approach form the optimal feature set for one-site p53 mutants. (B) The IFS curve for two-site p53 mutants. The peak of MCC is 0.341 with 52 features. The top 52 features derived by the CFS Subset Evaluator approach form the optimal feature set for two-site p53 mutants. (C) The IFS curve for three-site p53 mutants. The peak of MCC is 0.784 with 30 features. The top 30 features derived from the CFS Subset Evaluator approach form the optimal feature set for three-site p53 mutants. (D) The IFS curve for four-site p53 mutants. The peak of MCC is 0.916 with 15 features. The top 15 features derived from the CFS Subset Evaluator approach form the optimal feature set for four-site p53 mutants.

The predictor attributes were used to build individual predictors by inserting features in an incremental manner beginning at the first filtered attribute and proceeding till the attribute that generated the highest MCC was obtained. We tested each of the individual predictors and obtained the IFS results for all the filtered predictors. The Incremental Feature Selection for the site-specific mutation data was given as Table S3. The IFS Curves for the site-specific mutation data are portrayed in Figure 2A, 2B, 2C, and 2D respectively. The MCC of the site-two mutation data was compared using 3-fold cross –validation method. The optimal performance of the proposed predictor models is tabulated in Table 3.

**Table 3:** Optimal Performance of Novel Predictor Methods on Site-Wise P53 Mutants Transcriptional Activity.

*Site*	*Predictor Method*	*Algorithms Employed*	*Optimal no. of features*	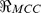	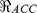	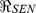	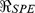
Site -1	Independent Predictor	CFS+ADBS	7	0.775	95.2	0.952	0.78
Site -2	Imbalanced Predictor	CFS+Random Committee	52	0.341	99.2	0.992	0.178
Site -3	Balanced Predictor	CFS+Bayes Network	30	0.784	89.3	0.893	0.894
Site -4	Balanced Predictor	CFS+Bayes Network	15	0.916	96.8	0.968	0.991
Site -5	Imbalanced Predictor	CFS+RCRT	1–154	0.655	87.5	0.875	0.625

### Performance Comparison of Proposed Predictors with Other Methods

We investigated the performance of Bayesian and Ensemble learning methods and found that a single technique did not generate optimal results on all site-specific mutation data with the CFS Subset attribute evaluator methods. So we attempted to identify the specific combination of attribute evaluator and prediction algorithm that generated optimal results with minimal features. The improved performance of our work was validated by the results of the previous work on predicting site-specific p53 mutant activity by Huang et al. [20]. The comparative performance of the classification algorithms on site-specific p53 mutation data is given as Table 4, Table 5, Table 6, Table 7 and Table 8 for one-, two-, three-, and four- and five-site mutation data. The Information Gain, Gain Ratio and Symmetric Uncertainty Attribute Evaluators used the Ranking method to generate the attribute evaluation results. With respect to the Independent and Balanced Predictor models, we considered all the ranked values for comparison. However for the two-site mutation data, since the features spanned large dimensions we set the information gain score to 0.02, gain ratio and symmetric uncertainty score to 0.05 to select the ranked attributes and the comparison among the methods was recorded accordingly. All the performance parameters were obtained by Jack-knife cross-validation approach for one-site, three-site, four-site and five-site mutants. However in order to reduce the memory effects and computational complexity, we used the three-fold cross-validation approach to compare the performance of two-site predictor models.

**Table 4:** Performance Comparison of Site-1 P53 Mutants Transcriptional Activity.

S.No	Attribute Evaluator	Prediction techniques	Features	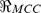	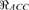	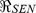	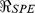
1	***CFS***	***Adaboost (Decision Stump)***	***11***	***0.616***	***90.3***	***0.903***	0.773
		Bayesian Network Learning		-0.087	82.3	0.823	0.122
		Random Committee		0.416	88.7	0.887	0.451
2	***Information Gain***	***Adaboost (Decision Stump)***	***19***	***0.688***	***93.5***	***0.935***	0.671
		Bayesian Network Learning		-0.087	82.3	0.823	0.122
		Random Committee		0	87.1	0.871	0.129
***3***	***Gain Ratio***	***Adaboost (Decision Stump)***	***19***	***0.688***	***93.5***	***0.935***	0.671
		Bayesian Network Learning		-0.087	82.3	0.823	0.122
		Random Committee		0.333	88.7	0.887	0.238
***4***	***Symmetric Uncertainty***	***Adaboost (Decision Stump)***	***19***	***0.688***	***93.5***	***0.935***	0.671
		Bayesian Network Learning		-0.087	82.3	0.823	0.122
		Random Committee		0.333	88.7	0.887	0.238

**Table 5:** Performance Comparison of Site-2 P53 Mutants Transcriptional Activity.

S.No	Attribute Evaluator	Prediction techniques	Features	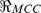	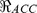	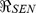	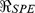
1	***CFS***	Adaboost (Decision Stump)	52	0	99.7	0.997	0.003
		Bayesian Network Learning		.162	97.2	0.972	0.475
		***Random Committee***		***0.341***	***99.2***	***0.992***	0.178
2	Information Gain	Adaboost (Decision Stump)	50	0	99.6	0.996	0.003
		Bayesian Network Learning		-0.001	96.5	.965	0.003
		Random Committee		0	99.7	0.997	0.003
3	Gain Ratio	Adaboost (Decision Stump)	40	0	99.7	0.997	0.003
		Bayesian Network Learning		0.13	98.8	.989	0.213
		Random Committee		.146	99.6	.996	0.073
4	Symmetric Uncertainty	Adaboost (Decision Stump)	40	0	99.7	0.997	0.003
		Bayesian Network Learning		.132	99.8	.998	0.231
		Random Committee		.159	99.6	.996	0.073

**Table 6:** Performance Comparison of Site-3 P53 Mutants Transcriptional Activity.

S.No	Attribute Evaluator	Prediction techniques	Features	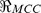	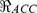	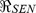	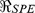
1	***CFS***	Adaboost (Decision Stump)	35	0.498	75	0.75	0.751
		***Bayesian Network Learning***		***0.745***	***87.5***	***0.875***	0.866
		Random Committee		0.57	78.6	0.786	0.788
2	Information Gain	Adaboost (Decision Stump)	417	0.451	73.2	0.732	0.705
		Bayesian Network Learning		0.358	67	0.67	0.689
		Random Committee		0.311	66.1	0.661	0.65
3	Gain Ratio	Adaboost (Decision Stump)	417	0.469	74.1	0.741	0.717
		Bayesian Network Learning		0.358	67	0.67	0.689
		Random Committee		0.311	66.5	0.665	0.65
4	Symmetric Uncertainty	Adaboost (Decision Stump)	417	0.469	74.1	0.741	0.717
		Bayesian Network Learning		0.358	67	0.67	0.689
		Random Committee		0.367	68.8	0.688	0.68

**Table 7:** Performance Comparison of Site-4 P53 Mutants Transcriptional Activity.

S.No	Attribute Evaluator	Prediction techniques	Features	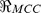	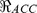	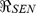	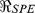
1	***CFS***	Adaboost (Decision Stump)	16	0.812	93.5	0.935	0.779
		***Bayesian Network Learning***		***0.91***	***96.8***	***0.968***	0.889
		Random Committee		0.392	80.6	0.806	0.539
2	Information Gain	Adaboost (Decision Stump)	73	0.812	93.5	.935	0.779
		Bayesian Network Learning		0.321	.774	.774	0.529
		Random Committee		0.354	80.6	0.806	0.438
3	Gain Ratio	Adaboost (Decision Stump)	73	0.812	93.5	0.935	0.779
		Bayesian Network Learning		0.321	.774	.774	0.529
		Random Committee		0.483	83.9	.839	0.548
4	Symmetric Uncertainty	Adaboost (Decision Stump)	73	0.812	93.5	0.935	0.779
		Bayesian Network Learning		0.321	.774	.774	0.529
		Random Committee		0.517	83.9	0.839	0.649

**Table 8:** Performance Comparison of Site-5 P53 Mutants Transcriptional Activity.

S.No	Attribute Evaluator	Prediction techniques	Features	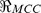	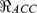	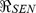	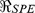
1	**CFS**	**Adaboost (Decision Stump)**	154	**0.655**	**87.5**	**0.875**	0.625
		Bayesian Network Learning		0	75	0.75	0.25
		**Random Committee**		**0.655**	**87.5**	**0.875**	0.625
2	Information Gain	Adaboost (Decision Stump)	154	**1**	**1**	**1**	1
		Bayesian Network Learning		0	75	0.75	0.25
		Random Committee		0.655	87.5	0.875	0.625
3	Gain Ratio	Adaboost (Decision Stump)	154	**1**	**1**	**1**	1
		Bayesian Network Learning		0	75	0.75	0.25
		Random Committee		0.655	87.5	0.875	0.625
4	Symmetric Uncertainty	Adaboost (Decision Stump)	154	**1**	**1**	**1**	1
		Bayesian Network Learning		0	75	0.75	0.25
		Random Committee		0.655	87.5	0.875	0.625

The Independent Predictor (IP) model utilized the CFS Subset Evaluator followed by the ABDS algorithm to obtain the optimal MCC. The algorithm was executed with default parameters with the number of iterations set to 100 to avoid over fitting of the data and obtain reliable results. Though the other attribute evaluator methods also showed promising results, the size of the feature set was taken into consideration to choose the most optimal approach. However the proposed approach equalled or bettered the other compared methods as depicted in Table S3 using Incremental Feature Selection. The Imbalanced Mutation Predictor (IMP) model utilized the CFS Subset Evaluator with RCRT algorithm to obtain the optimal MCC. The algorithm was run with default parameters and evaluated by 3-fold cross validation for two-site mutation data on account of large number of instances and increased computational complexity. The execution time of Jack-knife cross validation on site-two mutation data with default parameters was 24 hours and 17 minutes to validate the RCRT approach and reported an MCC of 0.293 with 52 features. The same predictor model was applied to five-site mutation data and generated an MCC of 0.655 with the smallest feature set comprising of 1 feature. However attributes generated by the Information Gain, Gain Ratio and Symmetric Uncertainty Attribute Evaluators generated a high MCC of 1 using ABDS algorithm with default parameters on the five-site mutation data. Since the results appeared to over fit the data on account of very small number of instances, the imbalanced predictor model was believed to be a more reliable technique. The Balanced Predictor (BP) model utilized the CFS Subset Evaluator with Bayesian Network Learning Algorithm and obtained an optimal MCC of 0.784 with 30 features on the three-site mutation data and an optimal MCC of 0.916 with 15 features on the site-four mutation data. The results were validated by Jack-knife cross-validation method. The feature set analysis of site-specific mutants is discussed below.

### Site –Wise Feature Set Analysis

**Figure 3:**
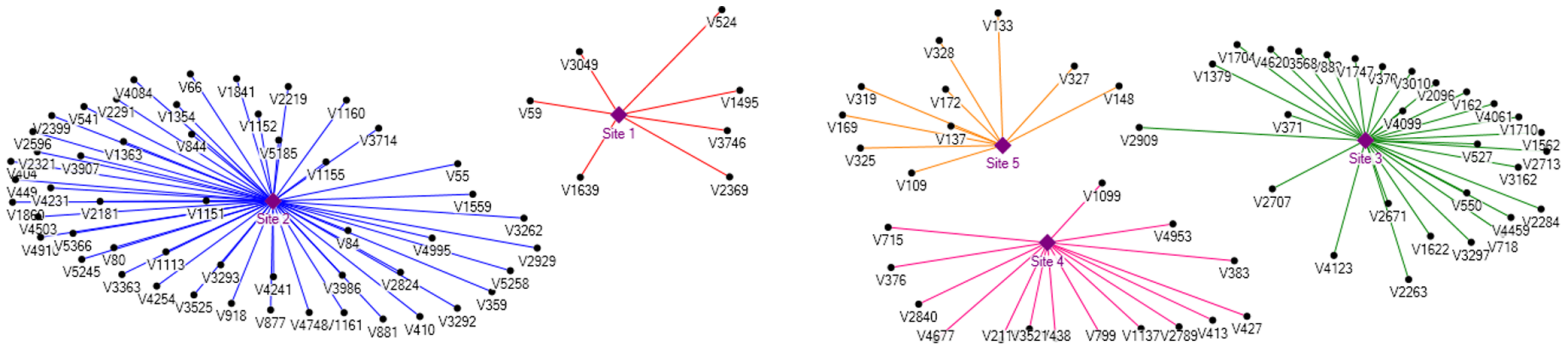
Site-wise Feature Relevance Graph. The sites are represented in purple as solid diamonds. The optimal features for each site are represented by directed edges from the site to the feature. Site-specific features are displayed in different colours.

On analysis of the feature sets that generated optimal results, it was concluded that the 2D features played a dominating role when compared to the 3D features and hence an in-depth analysis of 2D structural properties could provide novel insights into p53 functional mechanism. Site-1, Site-3 and Site-5 mutation data attained the optimal MCC with the inclusion of 2D features alone. However site-2 and site-4 mutation data attained the highest MCC only on inclusion of the 3D predictor features. We also attempted to explore the 2D and 3D features that were found relevant for the different site-wise subsets representing p53 transcriptional activity using structural properties and identify if there existed any common relevant features that deserved further attention. Hence we made use of visualization tool NodeXL available at [http://nodexl.codeplex.com/releases/view/96383] that generated the site-wise feature-activity relevance graph depicted in Figure 3 to represent the relevant features reported for each site. The visualization of the p53 site-wise feature relevance graph is shown in Figure 3. We used the top 10 features for the site-5 mutation data. The graph clearly depicts that not a single feature was commonly relevant to any of the sites. The features were mutually exclusive and hence we believed it was acceptable that any further investigations of the p53 functional activity would certainly warrant a site-wise analysis of structure and function.

### Comparison to Previous Work

The most recent and previously reported results of predicting p53 mutants transcriptional activity was stated by Huang et al., in 2011. The comparative performance to the previous work is depicted in Table 9. One-site mutation was optimally predicted at 0.678 MCC with 8 features whereas the proposed predictor model predicts at 0.775 MCC with 7 features while two-site mutation data was predicted at an optimal 0.314 MCC with 50 features while our approach attained an optimal prediction of 0.341 with 52 features. However they have excluded the memory effects of running Jack-knife cross validation on the 16376 records. Our results were drawn with 3-fold cross-validation that is reported to be a benchmark validation technique for large datasets [50]–[51] [73]. For the three-site mutation data, the proposed approach generated an optimal MCC of 0.784 with 30 features while the previous optimal MCC of 0.705 included 282 features. The four-site mutation data was predicted at 0.916 by our proposed approach with 15 features while the previous approach reported an optimal MCC of 0.907 with 25 features. Our findings agree with the previous results stating 2D features to be the major contributory factors to p53 mutant transcriptional activity prediction. The MCC and accuracy parameters of the predictor methods were found to be highly irrelevant in estimation of predictor performance of unbalanced datasets. Since this research was oriented towards both balanced and unbalanced datasets, MCC was utilized as the primary criterion for ranking the predictor models.

**Table 9:** Comparison to Previous Work on P53 Mutants Transcriptional Activity Prediction.

*S.No*	*Site*	*Previously Reported*		*Currently Reported*	
		*Optimal features*	*Optimal MCC*	*Optimal feature(s)*	*Optimal MCC*
1	One	8	0.678	7	0.775
2	Two	50	0.314	52	0.341
3	Three	282	0.705	30	0.784
4	Four	25	0.907	15	0.916
5	Five	Not Reported		1	0.655

## Discussion

### CFS Subset Vs mRMR Method

Previous work on prediction of p53 transcriptional activity made use of the Maximum Relevance and Minimum Redundancy (mRMR) approach in order to select the features most relevant to the target class and least redundant to one another [20]. The mRMR method ranked features based on the Mutual Information criterion [77]–[79]. In this study however we chose to investigate other possible feature selection algorithms for three main reasons: (i) Performance of the mRMR method has already been discussed in p53 transcriptional activity prediction [20]whereas this is the first study on utilization of CFS Subset evaluator and the other ranking methods (Information Gain, Gain Ratio and Symmetric Uncertainty) in p53 activity prediction (ii) Human intervention is required in deciding the feature subset size for the mRMR method [21]whereas in the CFS Subset method, the default parameters of Best First Search with a search termination threshold of five, generated the appropriate and relevant feature subset [35] (iii) It is evident from the work on p53 transcriptional activity prediction by Huang et al. [20] where roughly 100 to 1000 ranked features from the mRMR method were included for the Incremental Feature Selection process to obtain optimal results whereas in this investigation the feature subset size returned by the CFS Subset Evaluator on the same datasets was of considerably smaller dimension thus entailing less human effort and time while generating improved results. Moreover we believed the CFS Subset Evaluator would certainly prove to be an effective algorithm in other biological data prediction also and hence propose a reasonably acceptable alternative to the mRMR method. Further extensions to this work would involve investigating the use of this novel methodology in DNA and protein sequence analysis.

### Influence of Structure on P53 Function

This research has clearly revealed the contribution of the structural features in predicting p53 transcriptional activity. Previous authors [20] [25]–[26] [30] [44] have stated that structural features played a dominant role in p53 status prediction. However this study has clearly portrayed through the use of computational techniques that 2D properties played the most contributing role in P3 transcriptional activity prediction. A characteristic feature of the p53 mutational map is the frequency of missense point mutations [74]–[75]. Structural studies have revealed a higher concentration of amino acid residues pertaining to the mutation hot spots of p53 within the central region (residues 102–292), encoding the central DNA binding domain of the protein, and a trivial number of p53 mutations in the regulatory domains (N terminus, residues 1–99; C terminus, residues 301–393) [74]–[76]. This drives research focus towards concluding that intense analysis of p53 structure could reveal yet unknown facts on p53 activity thus leading to novel therapeutic solutions.

### Rewards of Computational Strategies

Previous work on p53 Mutants and related studies have brought to light the hurdles encountered in in-vitro experimentation with mutation data in view of the resources, labour and time involved, but with irresolute rewards [2] [6] [13] [16] [20] [30]–[32]. On the contrary, computational strategies and algorithms expend comparatively less time, resources and labour with a clear idea of expected end results [19]–[21] [25]–[28]. The broad goal of this work was to provide an influential assessment of the functional activity of p53 cancer mutants and their secondary-site suppressor mutations through the use of computational techniques. A functional census of suppressor mutations for p53 cancer mutants was believed to appreciably further existing knowledge of p53 rescue mechanisms [32] [74] [76]. Knowledge of possible regions of the p53 core domain that generated stability when altered provided insights in detecting probable alteration sites for small molecules. The methodology could be generalized to other mutational systems where mutants needed to be classified as functional/non-functional. Moreover computational classifiers that predicted mutant function would allow experimentalists to map structure/function relationships for proteins in other mutation-related diseases.

With the advances in technology and their applications in the field of biology and medicine influencing the focus of research in remarkable ways, we believed research and analysis of the effects of computational methods on biological data analysis was certainly an essential breakthrough. However a limiting factor in computational analysis was the measure of time spent on preparing biological data for process on software tools. Efficient data pre-processing techniques specific to biological data could spur great opportunities for further investigation in the field of Bioinformatics and Computer Science.

### Conclusion

Intense research on p53, its structure, function and therapeutic strengths has drawn the attention of researchers from varied domains that include medical science, technology and informatics. This research was focused on revealing the significance of computational techniques in predicting the most optimal set of structural features that contributed predominantly to designating the nature of p53 transcriptional activity. We compared the performance of four feature evaluator and three classification techniques to determine the optimal set of features that predicted p53 activity with higher MCC. Our findings revealed the optimal MCC in prediction of p53 transcriptional activity with the most predictive feature set for each site-specific mutation subsets. Moreover visualization of the site-specific relevant features indicated that the contributing features were mutually exclusive for each site and appeared only on a section of the mutation sites. This could be attributed to the fact that unselected mutations contributed nothing to the p53 activity while selected features played the crucial role in regulation of p53 activity. We also warrant the fact that the 2D structural properties deserved more attention and further analysis of their influence on p53 mutations could reveal latent facts on the underlying mechanism of p53 and provide novel and informative insights into p53 transcriptional activity and their restoration.

## Supporting Information

Table S1 **Description of the 2D and 3D structural properties of p53 mutants.**
(XLS)

Table S2 **CFS Subset Attribute Evaluator results for site-specific p53 mutant data.**
(XLS)

Table S3 **Incremental Feature Selection Results of site-specific p53 mutant data.**
(XLS)
